# Effect of an exercise training intervention with resistance bands on blood cell counts during chemotherapy for lung cancer: a pilot randomized controlled trial

**DOI:** 10.1186/2193-1801-3-15

**Published:** 2014-01-08

**Authors:** Kristina H Karvinen, David Esposito, Thomas D Raedeke, Joshua Vick, Paul R Walker

**Affiliations:** School of Physical and Health Education, Nipissing University, 100 College Drive, North Bay, Ontario P1B 8 L7 Canada; Department of Kinesiology, East Carolina University, Minges Coliseum, Greenville, North Carolina 27858 USA; Department of Internal Medicine, Division of Medical Oncology, Brody School of Medicine at East Carolina University, Greenville, North Carolina 27834 USA

**Keywords:** Lung cancer, Exercise, Blood counts, Chemotherapy

## Abstract

**Purpose:**

Chemotherapy for lung cancer can have a detrimental effect on white blood cell (WBC) and red blood cell (RBC) counts. Physical exercise may have a role in improving WBCs and RBCs, although few studies have examined cancer patients receiving adjuvant therapies. The purpose of this pilot trial was to examine the effects of an exercise intervention utilizing resistance bands on WBCs and RBCs in lung cancer patients receiving curative intent chemotherapy.

**Methods:**

A sample of lung cancer patients scheduled for curative intent chemotherapy was randomly assigned to the exercise intervention (EX) condition or usual care (UC) condition. The EX condition participated in a three times weekly exercise program using resistance bands for the duration of chemotherapy.

**Results:**

A total of 14 lung cancer patients completed the trial. EX condition participants completed 79% of planned exercise sessions. The EX condition was able to maintain WBCs over the course of the intervention compared to declines in the UC condition (*p* = .008; *d* = 1.68). There were no significant differences in change scores in RBCs.

**Conclusions:**

Exercise with resistance bands may help attenuate declines in WBCs in lung cancer patients receiving curative intent chemotherapy. Larger trials are warranted to validate these findings. Ultimately these findings could be informative for the development of supportive care strategies for lung cancer patients receiving chemotherapy.

**Trial registration:**

Clinical Trials Registration #: NCT01130714.

## Background

Chemotherapy for lung cancer can have a detrimental effect on both red blood cell (RBC) and white blood cell (WBC) counts due to bone marrow suppression. Declines in WBC subsets can result in delays in chemotherapy and reductions in dosage (Khan et al. 2008) and increased risk of infection, mortality and hospital admissions (Klastersky et al. 2009). Similarly, declines in RBC indices can exacerbate fatigue, reduce quality of life, result in delays in treatment, and have a negative impact on survival (Hauser et al. 2006; Pirker et al. 2003).

Although a large body of research has focused on the role of physical exercise as a potential supportive care strategy for cancer survivors (Speck et al. 2010), relatively little research has examined its effect on hematologic outcomes, especially in lung cancer patients and survivors. In general populations exercising is associated with an increase in circulating WBCs (Freidenreich and Volek 2012). Although WBCs typically return to baseline hours after an acute bout of exercise in healthy populations, it is unclear what the effect of regular exercise may be in individuals being treated for cancer whose levels are compromised as a result of adjuvant therapies. Of the research to date, significant improvements in WBC subsets have been found following an exercise program in patients receiving high dose chemotherapy followed by autologous peripheral blood stem cell transplantation (Dimeo et al. 1997b) and in prostate cancer survivors receiving androgen deprivation therapy (Galvao et al. 2008).

Exercise has also been found to have a positive effect on RBC indices in human and animal models (Hu et al. 2011). Although not fully understood, the benefits of exercise on RBC counts may be at least partially explained through the intertwining of the processes of ossification and hematopoiesis (Hu et al. 2011). Through high force and/or impact exercise, bone formation is stimulated resulting in a simultaneous rise in RBC indices (Guadalupe-Grau et al. 2009). Although the exact mechanism has yet to be discovered, a recent study found evidence of crosstalk between osteoblasts and hematopoietic stem cells which may account for an increase in both RBC indices and biomarkers of bone formation (Hu et al. 2008; 2011). Only a paucity of research has examined the effect of an exercise intervention on RBC indices in cancer survivors, including in patients receiving high dose chemotherapy (Dimeo et al. 1997a), breast cancer survivors receiving radiation therapy (Drouin et al. 2006) and chemotherapy (Dolan et al. 2010). Results were generally similar to work in other populations indicating a favourable effect of exercise on RBCs.

In this paper we add to the small body of existing research examining the effect of exercise on hematologic outcomes in cancer patients. Moreover, we focus on lung cancer patients on chemotherapy, a group that has only recently been studied in the exercise domain (Adamsen et al. 2012; Hoffman et al. 2013; Kuehr et al. 2013; Quist et al. 2012; Temel et al. 2009). The purposes of this pilot trial were to examine the effects of an exercise training intervention utilizing resistance bands on WBC and RBC counts in lung cancer survivors receiving curative intent chemotherapy compared to a usual care control group. It was hypothesized that lung cancer survivors in the exercise (EX) condition would maintain WBC and RBC counts during chemotherapy compared to declines in the usual care (UC) condition.

## Methods

### Trial design

The study was a two-armed parallel design prospective randomized controlled trial. Study participants were stratified by sex and type (non-small cell vs. small cell) and randomly assigned with a computer-generated program to either the EX or UC conditions in a 1:1 ratio in blocks of 4. An outside research assistant created the allocation sequence and assembled the results into a series of sequentially numbered opaque envelopes. The results were concealed from the research team until the next consecutive envelope was opened by a research assistant in the presence of the study participant.

### Participants and setting

The study was approved by the University and Medical Center Institutional Review Board at East Carolina University. Participants were recruited from a cancer center in Eastern North Carolina and gave written and informed consent in English before commencing the trial (Clinical Trials Registration number NCT01130714). Eligibility criteria included (a) histologically confirmed diagnosis of non-small cell or small cell lung cancer, (b) eligible for chemotherapy with curative intent, (c) 21 years of age or older, and (d) approved to participate in trial by treating oncologist. Individuals were excluded if they had already commenced chemotherapy to treat lung cancer. All baseline and post intervention assessments were completed at the cancer center.

### Exercise program

Participants randomized to the EX condition participated in the exercise program for the duration of their chemotherapy starting one week after the first cycle of chemotherapy began until 3 weeks after the last cycle (approximately 4 cycles lasting a total 12 weeks). The exercise program consisted of an exercise routine with resistance bands that consisted of total body exercises three times per week with at least one day of rest between sessions. Exercises simulated traditional resistance exercises with free weights and included biceps curls, shoulder pull backs, seated rows, triceps extensions, chest press, leg extensions, hip flexion, hip abduction, hip adduction, heel raises, and chair squats. Resistance bands are portable, inexpensive, can be used for a variety of total body exercises, and have been used successfully with older populations (Egana et al. 2010) and in a recent trial of cancer survivors receiving high dose chemotherapy and hematopoietic stem cell transplantation (Hacker et al. 2011). One weekly exercise session with the resistance bands was completed under the supervision of an exercise trainer at the treating cancer center while the other two sessions per week were performed at the participant’s home with guidance from a guide booklet and DVD developed for the trial. Exercise sessions were not completed on days when chemotherapy was administered. The exercise routine consisted of warm-up exercises and stretches followed by 12 total body resistance exercises working major muscle groups for two sets of 15 repetitions. The full routine took approximately 30 minutes to complete. All exercises were chair based and developed from an existing program utilized for cardiac and pulmonary patients by an exercise physiologist. Participants were asked to maintain sufficient tension in the bands to find it challenging to complete the second set of repetitions. The UC condition continued with usual care and received a complimentary training session, guide booklet and DVD after completion of all post intervention measures.

### Primary and outcome measures

Primary outcome measures (i.e., WBC and RBC counts) were assessed at baseline (within one week of commencing the first cycle of chemotherapy) and post intervention (three to four weeks following the last delivery of chemotherapy). EX condition participants were instructed to not complete any exercise sessions within 24 hours of their scheduled post intervention blood draw.

*White and red blood cell counts* were determined by routine CBC at the cancer center laboratory. Results were obtained from patient charts.

### Other measures

*Demographic and medical information* were assessed from patient records (cancer type, stage, treatment received) and questionnaire (date of birth, race, height, weight, smoking status, number of comorbidities).

*Exercise level* at baseline was determined by the Leisure Score Index (LSI) of the Godin Leisure-Time Exercise Questionnaire GLTEQ; (Godin et al. 1986). The LSI assesses the average frequency of light, moderate and vigorous exercise in a typical week in the past month. As in previous investigations (Vallance et al. 2007), a modified version was used that also measures duration of exercise. The LSI has been found to be valid and reliable with concurrent validity coefficients of .32 compared to an accelerometer and .56 when compared to VO_2max_ and a one-month test-retest reliability of .62 (Jacobs et al. 1993). For each intensity level (i.e., light, moderate, vigorous), the number of sessions per week of exercise was multiplied by the average number of minutes per session to obtain weekly measures of exercise minutes at each intensity. Two separate variables for average minutes of moderate intensity exercise and average minutes of vigorous intensity exercise were calculated. An additional composite measure of moderate-to-vigorous exercise minutes in a typical week in the past month was created by multiplying the vigorous intensity minutes variable by two and adding it to the moderate intensity variable [i.e., (weekly vigorous minutes × 2) + (weekly moderate minutes)] as per the US Department of Health and Human Services’ (Physical Activity Guidelines for Americans 2008 physical activity categories. Study participants were classified as “exercisers” or “non-exercisers” based on whether or not they were meeting American College of Sports Medicine public health recommendations for exercise (i.e., 150 weekly minutes of moderate and vigorous intensity exercise combined or 60 weekly minutes of vigorous intensity exercise; Haskell et al. 2007) according to the exercise variables described above. To remain consistent with public health recommendations for exercise, light intensity exercise minutes were not included in the exercise measures (Haskell et al. 2007).

*Adherence to the EX intervention* was assessed from weekly exercise logs. EX condition participants were asked to record each exercise session in their logs and return them to the exercise trainer during their weekly supervised exercise session.

### Sample size calculation and statistical analyses

At 0.67 power, 12 participants were required to detect a large effect (*d* = 1.0) at α = .05. Comparisons between baseline demographic and medical characteristics and baseline outcome measures were examined by independent samples t-tests for continuous data and chi-square analyses for categorical data. Independent samples t-tests were used to calculate differences between the EX and UC conditions for WBC and RBC at post-intervention. Differences in change scores between groups were examined using independent samples t-tests. Effect sizes (*d*) were calculated by dividing the difference of the means by the pooled standard deviation (Cohen 1992). An effect size of 0.2 was considered a small effect, 0.5 a medium effect, and 0.8 and greater a large effect (Cohen 1992). Because of the small sample size and the difficult to treat population, a modified intention-to-treat analysis was used for all analyses. The modification included exclusion of participants from analyses if they had suffered a major adverse event during the course of the intervention or did not complete any scheduled exercise sessions (supervised or home-based). All other participants were included in the analyses including those with some missing baseline demographic data (see Table 1).

**Table 1:** **Baseline demographic and medical characteristics**

Variable	Overall	EX	UC	P
Age (years; N = 14)	58.8 (12.9)	64.2 (12.9)	55.8 (12.6)	.258
Sex (N = 14)				
Male	10	4	6	.597
Female	4	1	3	
Race (N = 14)				
Caucasian	11	4	7	.923
African American	3	1	2	
Education (N = 10)				
High school or less	7	3	4	.490
Some post secondary school	3	2	1	
Body mass index (kg/m^2^) (N = 14)	31.5 (7.4)	29.7(4.3)	33.4 (9.8)	.462
Smoking status (N = 14)				
Never smoked	2	1	1	.375
Ex-smoker	8	4	4	
Current smoker	2	0	2	
Quit during treatment	2	0	2	
Exercise at baseline (N = 11)				
Exerciser	2	0	2	.114
Non-exerciser	9	5	4	
MV exercise minutes^a^	114.2 (232.1)	8 (11.0)	220.4 (304.9)	.158
Moderate exercise minutes^b^	47.3 (79.1)	8 (11.0)	80 (98.0)	.140
Vigorous exercise minutes^c^	6.4 (18.0)	0 (0)	11.7 (24.0)	.310
Number of comorbidities (N = 14)	1.7 (1.1)	2.2 (0.8)	1.2 (1.1)	.143
Type of lung cancer (N = 14)				
Adenocarcinoma	5	1	4	
Squamous	6	4	2	
Large cell	1	0	1	
Small Cell	1	0	1	
NSCLC: Poorly differentiated	1	0	1	
Stage (N = 14)				
I	1	0	1	
IIB	2	1	1	
IIIA	8	2	6	
IIIB	2	2	0	
IV	1	0	1	
Chemotherapy (N = 14)				
Cisplatin/Irinotecan	7	2	5	
Cisplatin/Pemetrexed	2	0	2	
Carboplatin/Irinotecan	2	1	1	
Paclitaxel/Bevacizum	1	1	0	
Carboplatin/Pemetrexed	1	0	1	
Taxol/Irinotecan	1	1	0	
Radiation Therapy (N = 14)				
None	1	1	0	
50.4 Gy	1	0	1	
60 Gy	4	1	3	
66 Gy	7	3	4	
PCI^d^	1	0	1	
Surgery (N = 14)				
Inoperable	5	2	3	
Preoperative	7	3	4	
Postoperative	2	0	2	

^a^Moderate-to-vigorous intensity exercise minutes in a typical week in the month before baseline.

^b^Moderate intensity exercise minutes in a typical week in the month before baseline.

^c^Vigorous intensity exercise minutes in a typical week in the month before baseline.

^d^Prophylactic Cranial Irradiation.

## Results

**Figure 1:**
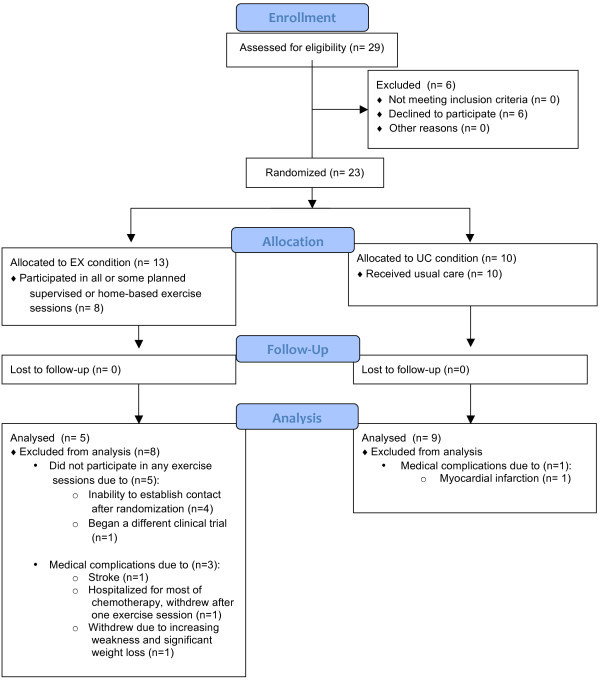
**Flow diagram Of participants through the trial.**

Participants were recruited for the study between January 2010 and September 2011 (Figure 1). Of the 29 deemed eligible, 23 were recruited for the study indicating a 79% recruitment rate. A total of 61% (14/23) of participants were included in the analyses. The main reason for exclusion from analyses was failure to reach the participant again after multiple attempts for contact resulting in incompletion of all exercise sessions (both supervised and home-based; n = 5). Significantly more EX condition participants were not included in the analyses compared to the control condition (*p* = .012). There were no significant differences in demographic and medical characteristics between the groups at baseline (Table 1). Although not statistically different, the UC condition had a higher number of average weekly exercise minutes at baseline compared to the EX condition due to two UC participants being found to be regular exercisers. We re-analyzed the data with these two participants excluded and found almost identical findings to the original analyses with the full sample. Thus we report the analyses with the full data in this manuscript in order to maintain a larger sample. The mean length of the intervention was 12 to 16 weeks. EX condition participants completed a total of 79% of planned exercise sessions (both supervised and home-based).

### Demographic and medical variables

Details of demographic and medical variables are displayed in Table 1. In summary, the average age of the sample was 59 years, the majority of participants were male, Caucasian and current or former smokers. All participants who received radiation therapy did so concurrently with chemotherapy with the exception of one UC participant who received prophylactic cranial irradiation following chemotherapy.

### Changes in outcome measures

Baseline, post intervention and change scores for RBCs and WBCs are displayed in Table 2. No significant differences were found between groups on either outcome measure at baseline. At post intervention the EX condition indicated significantly higher WBC scores compared to the UC condition (*p* = .043; *d* = 1.19) and no significant differences in RBC (*p* = .147; *d* = .82). Based on the analysis of differences in change scores, WBC declined significantly less in the EX condition compared to the UC condition over the course of the intervention (*p* = .008) indicating a large effect size (*d* = 1.68). There were no significant differences in change scores in RBC between the EX and UC conditions and the effect size was small (*p* = .555; *d* = .32).

**Table 2:** **Effects of exercise training on outcome variables**

Outcome	Baseline				Post Intervention				Change			
	Mean	SD	***P***	***d***	Mean	SD	***P***	***d***	Mean	SD	***P***	***d***
WBC (×10^3^/μL)												
EX	7.90	1.21	.167	0.93	7.86	4.40	.043	1.19	-.04	4.67	.008	1.68
UC	9.46	2.16			4.18	1.75			-5.28	1.56		
RBC (million cells/μL)												
EX	4.17	.38	.533	0.40	2.85	.61	.147	0.82	-1.32	.83	.555	.32
UC	4.38	.65			3.27	.41			-1.11	.51		

### Adverse events

There were three adverse events in the EX condition and one in the UC condition. In the EX condition one participant suffered a stroke (completed eight exercise sessions prior to the stroke), one was hospitalized for most of the intervention (completed one exercise session prior to hospitalization) and one withdrew due to increasing weakness and weight loss (completed eight exercise sessions before withdrawing). In the UC condition one participant suffered a myocardial infarction during the course of chemotherapy and was withdrawn from analyses.

## Discussion

This pilot trial provides novel information about the effect of an exercise program utilizing resistance bands on blood counts in lung cancer survivors receiving curative intent chemotherapy. In partial support of our hypotheses, we found a significant difference in changes in WBC in favor of the EX condition. There were however no significant differences in changes in RBC between the EX and UC conditions.

The positive effect of exercise training on WBC in this pilot trial is in line with past research that suggested that participation in an exercise program may improve WBC and subsets in cancer survivors during adjuvant therapy (Dimeo et al. 1997b; Fairey et al. 2005; Galvao et al. 2008). In a randomized study of 70 patients with solid tumours receiving high dose chemotherapy followed by autologous peripheral blood stem cell transplantation, (Dimeo et al. 1997b) found that two weeks of daily “biking” with a bed ergometer significantly reduced duration of neutropenia compared to a usual care control condition. Moreover, multiple regression analyses found that the greatest predictor of neutropenia duration was exercise training when examined with other variables such as age, body mass index, total dose of carboplatin, physical performance at baseline, and number of reinfunded blood stem cells (Dimeo et al. 1997b). In a subsequent study of a sample of 10 prostate cancer survivors receiving androgen deprivation therapy, (Galvao et al. 2008) found a significant increase in lymphocytes from baseline after 10 weeks of twice weekly resistance training possibly due to improved immune surveillance in response to resistance training. Although more research is needed to substantiate these conclusions, our findings, along with that of past research, suggest exercise may have a positive effect on WBCs and subsets in cancer patient populations receiving adjuvant therapies.

The failure of our trial to show differences in changes in RBC counts between the EX and UC conditions was unexpected and inconsistent with past research in cancer survivors. For example, in a pilot trial of 16 cancer patients recently completing high dose chemotherapy, six weeks of treadmill walking resulted in better improvements in haemoglobin concentrations compared to a usual care control group (Dimeo et al. 1997a). Similarly, a seven week long walking program, three to five times per week for a duration of 20 to 45 minutes, improved RBC counts in a sample of breast cancer survivors compared to a placebo stretching group (N = 20; Drouin et al. 2006). One study, however, indicated no effect of 17 weeks of three times weekly aerobic or resistance exercise training on haemoglobin in breast cancer survivors on chemotherapy compared to a control group (N = 242), although significant positive correlations were found between haemoglobin and changes in aerobic fitness (Dolan et al. 2010).

The non significant difference in change scores between groups in RBC counts in our study may be related to several factors. First, it is possible that an exercise program utilizing resistance bands is not as effective for improving RBC outcomes as other forms of exercise. In both the two trials that resulted in improvements in RBC indices (Dimeo et al. 1997a; Drouin et al. 2006) the intervention consisted of a walking program while the other trial that indicated favourable RBC outcomes related to exercise involved an aerobic training program utilizing gym equipment (e.g., treadmills, exercise bikes). Although resistance training has been found to be effective in improving RBC indices in general populations (Hu et al. 2011), it is possible that resistance bands, especially when utilized by an extremely deconditioned population, do not cause sufficient force and impact to stimulate bone formation and subsequent hematopoiesis. Moreover, it is possible that an aerobic training component is necessary to elicit changes in RBC in cancer populations. It is also important to note that some exercise trials in other populations, such as chronic kidney patients (Chen et al. 2010) and adolescents (Ulrich et al. 2010) did not result in significant changes in RBCs, and that high volumes of endurance training typically result in decreases in RBCs (Mairbaurl 2013). Nonetheless, trials with larger sample sizes, different exercise modalities or means of objectively measuring exercise intensity may be needed to determine if exercise can have an effect on RBC counts in lung cancer populations.

The exercise program used for the study was found to be safe and possibly feasible for lung cancer patients that initiated the program. Although several adverse events were reported during the course of the intervention none were believed to be related to the exercise program. This finding is in line with past research that has found exercise to be generally safe for cancer survivors, even during treatment and recovery (Schmitz et al. 2010). Moreover, EX condition participants were able to complete a relatively high proportion of planned exercise sessions (79%), suggesting a three times weekly exercise program with resistance bands may be feasible for this population of lung cancer patients. Given lung cancer patients frequently indicate low aerobic capacity and limited mobility, training with resistance bands may be a more viable mode of exercise compared to aerobic training (e.g., walking or other cardio programs). Resistance bands may also be appropriate for lung cancer populations as all exercises can be completed from a seated position, tension can be adjusted to accommodate a wide range of abilities, and the entire program is portable enough to be used as an economical home based exercise program. Future research examining the utility of resistance bands as a part of exercise programming in lung cancer and other deconditioned patient populations is warranted to fully determine its potential use in supportive care.

The strengths of our trial include the high recruitment rate (79%), the use of a weekly supervised exercise session as part of the protocol, and the relatively high adherence rate to the program (79%) by the EX condition. The limitations include the small, heterogeneous sample, differential exclusion from analyses in the EX condition, and missing data on some baseline variables for some participants.

## Conclusion

In summary, this pilot trial suggests that exercise training utilizing resistance bands may attenuate declines in WBC but not RBC counts in lung cancer survivors receiving curative intent chemotherapy. Moreover, the exercise program utilized may be safe and feasible for this population. Cancer care providers may be advised to consider chair based exercise programs with resistance bands as a potential supportive care strategy for lung cancer survivors. Future large scale trials are needed to further validate these results in both lung cancer and other cancer populations.
